# Neglected wild life: Parasitic biodiversity as a conservation target^☆^

**DOI:** 10.1016/j.ijppaw.2013.07.002

**Published:** 2013-08-02

**Authors:** Andrés Gómez, Elizabeth Nichols

**Affiliations:** a American Museum of Natural History, Central Park West at 79th Street, New York, NY 10024, USA; b Lancaster Environment Centre, Lancaster University, Lancaster LA1 4YQ, UK; c Department of Ecology, Institute of Bioscience, University of São Paulo, 05508-900 São Paulo, SP, Brazil

**Keywords:** Macroparasite, Microparasite, Co-Extinction, Dependent taxa, Intrinsic value, Ecosystem service, Conservation strategy

## Abstract

Parasites appropriate host resources to feed and/or to reproduce, and lower host fitness to varying degrees. As a consequence, they can negatively impact human and animal health, food production, economic trade, and biodiversity conservation. They can also be difficult to study and have historically been regarded as having little influence on ecosystem organization and function. Not surprisingly, parasitic biodiversity has to date not been the focus of much positive attention from the conservation community. However, a growing body of evidence demonstrates that parasites are extremely diverse, have key roles in ecological and evolutionary processes, and that infection may paradoxically result in ecosystem services of direct human relevance. Here we argue that wildlife parasites should be considered meaningful conservation targets no less relevant than their hosts. We discuss their numerical and functional importance, current conservation status, and outline a series of non-trivial challenges to consider before incorporating parasite biodiversity in conservation strategies. We also suggest that addressing the key knowledge gaps and communication deficiencies that currently impede broad discussions about parasite conservation requires input from wildlife parasitologists.

## Highlights

• Parasites are extremely diverse and ubiquitous in natural ecosystems.
• They have critical roles in ecological and evolutionary processes.
• Many parasite species are believed to be threatened or already extinct.
• All arguments for the conservation of any species apply equally well to parasites.
• Parasite conservation can be a very challenging endeavor.

## Graphical abstract

**Figure:**
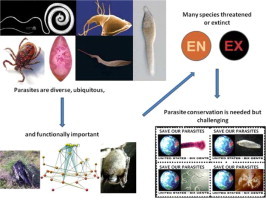

## 1 Introduction

Parasites have few friends. In the vernacular, the term “parasite” connotes free riders and slimy creatures. In nature, they are difficult to study due to their small size, complex life cycles, and generalized taxonomic impediments. In wildlife biology, parasites have traditionally been either ignored because quantifying their effects on host species is challenging, or antagonized because of the inherent harm they cause their hosts. Many human parasites, often zoonotic, carry important costs that result in morbidity, mortality, and negative effects on the economy (Gallup and Sachs, 2001; Gazzinelli et al., 2012). Wildlife parasites in particular, represent the majority of zoonotic emerging pathogens of humans (Taylor et al., 2001). Animal parasites also impact food security and incomes through their deleterious influences on livestock (Cleaveland et al., 2001). Finally, disease can affect conservation efforts, acting as a contributing threat in the endangerment of wildlife hosts, and occasionally causing severe population declines (de Castro and Bolker, 2005; Blehert et al., 2009). For all these reasons it is not surprising that parasites are generally viewed through the lens of either direct antagonism or patent disregard.

As a consequence, the maintenance of parasitic biodiversity has not historically been a conservation priority (Gompper and Williams, 1998; Dunn et al., 2009; Griffith, 2012). The stated goal of the field of conservation biology is to maintain biodiversity, including the evolutionary processes that drive and sustain it (Meffe et al., 2006). Yet to ignore the conservation of parasites is to ignore the conservation status of the majority of life on Earth, as parasitism represents the most common consumer strategy on the planet (Poulin and Morand, 2000; Dobson et al., 2008). It also means neglecting a fundamental biological relationship, as infection is fundamental to the ecological and evolutionary drivers of biological diversity and ecosystem organization (Marcogliese, 2004).

Here we argue that wildlife parasites should be considered meaningful conservation targets no less relevant than their hosts. We discuss their numerical and functional importance, current conservation status, and outline a series of non-trivial challenges to consider before incorporating parasite biodiversity in conservation strategies. We use the term “parasite” to refer to both micro and macroparasites. This diverse and multiphyletic group is united by their appropriation of resources from a host in some part of their life cycle. This appropriation creates direct fitness costs to host individuals, although the magnitude of said costs is highly variable and often context-dependent. Despite the increasing visibility of parasite conservation in the scientific literature (Gompper and Williams, 1998; Windsor, 1998; Gómez et al., 2012), this topic has seldom been addressed with specific reference to wildlife parasites. Here we focus on parasites of wildlife and the roles of wildlife parasitologists in discussions about parasite conservation.

## 2 Is the host-parasite relationship important?

Wildlife parasite studies have traditionally focused on the documentation of parasitic communities in host populations, surveillance for parasitic organisms of animal or human health relevance, or assessments of disease risk to long-term host persistence (Riley et al., 2004; Clifford et al., 2006; Pedersen et al., 2007; Hamer et al., 2012). More rarely are they concerned with the ecological and evolutionary ramifications of host-parasite associations (Gompper and Williams, 1998). However, recent research suggests host-parasite relationships are a fundamentally important driver of ecological structure and function. Parasites are a ubiquitous component of ecosystems in terms of species diversity (Poulin and Morand, 2004), biomass (Kuris et al., 2008), and relevance in food webs (Amundsen et al., 2009; Dunne et al., 2013).

By extracting resources from their hosts, parasites force them to alter their energy balances (Thomas et al., 2009) consequently influencing host fitness even in the absence of clinical signs of infection (Hudson et al., 2002). The resulting impacts of parasitism on host reproductive rate (Schwanz, 2008), growth (Gorrell and Schulte-Hostedde, 2008), movement, and survival (Robar et al., 2010) translate into influences on community and ecosystem organization. At small spatial scales, the differential effects of infection of generalist parasites can modulate competitive interactions. For example, parapoxvirus-mediated apparent competition likely explains the ecological success of introduced gray squirrels (*Sciurus carolinensis*) in the United Kingdom (Tompkins et al., 2002). Nematodes can modulate the coexistence (or lack thereof) of sympatric bird species (Tompkins et al., 2001), and meningeal worm (*Parelaphostrongylus tenuis*) favor white-tailed deer (*Odocoileus virginianus*) in habitats deer share with elk (*Cervus elaphus*) (Bender et al., 2005). Infection can also affect reproductive behaviors and output, for example causing abortion or sterility. In the most extreme case, parasitic castrators divert the host’s metabolism for their own reproductive success, driving changes in host density and maturation rates (Lafferty and Kuris, 2009).

Parasites can also shape patterns of animal distribution and density at larger spatial scales, as seen in the introduction and subsequent removal of the rinderpest virus in East Africa, which dramatically impacted ecosystem structure by influencing ungulate population densities (Thomas et al., 2005). The impacts of rinderpest infection over large-scale ecosystem processes (e.g. wildfire dynamics and the ecology of tree species) are still apparent across the Serengeti ecosystem (Holdo et al., 2009). Parasites are also natural selection agents influencing a variety of host attributes, from phenotypic polymorphism and secondary sexual characters, to the maintenance of sexual reproduction (Wegner et al., 2003; Lively et al., 2004; Blanchet et al., 2009). These effects ultimately drive biological diversification, through influencing host reproductive isolation and speciation (Summers et al., 2003).

Finally, recent discussions of the importance of parasites in food webs (Lafferty et al., 2008a; Britton, 2013; Dunne et al., 2013); as modulators of host behavior (Barber et al., 2000; Lefevre et al., 2009), drivers of community composition (Fenton and Brockhurst, 2008), competitive interactions and biological invasions (Hatcher et al., 2006, 2012; Dunn et al., 2012); and as selective agents (Summers et al., 2003; Nunn et al., 2004), provide multiple lines of evidence for the ecological and evolutionary relevance of parasitic biodiversity.

## 3 Are wildlife parasites endangered?

In the conservation literature, parasites are most often viewed as threats to their hosts (Nichols and Gómez, 2011), infection often understood as a sign of ecosystem disturbance (Patz et al., 2004), and the loss of wildlife seen as a driver of disease amplification (Randolph and Dobson, 2012). Recent research has shown that most human emerging diseases have a zoonotic reservoir, that reservoirs are most often wildlife species (Jones et al., 2008), and that anthropogenic disturbance is commonly associated with human and wildlife disease emergence events (Daszak et al., 2000). Particularly given the media attention paid to emerging zoonotic disease, it is possible that we live in an age characterized by a generalized perception that parasites must be controlled rather than conserved.

However, parasites are not immune to the threats that affect free-living species and our current biodiversity crisis may well be primarily characterized by the loss of affiliate species (Dunn et al., 2009). Reports of pandemics and emerging disease illustrate one of the consequences of global environmental change but do not preclude the fact that many parasite species are also threatened by it. We now know that ecosystem disturbance creates risks for parasite persistence (Hudson et al., 2006; Lafferty, 2012). For example, land-use change and pollution can both reduce the abundance and diversity of parasite species (Lafferty, 1997; Huspeni and Lafferty, 2004; Bradley and Altizer, 2007). Climate change can restrict parasite transmission (Afrane et al., 2012) and lead to phenological mismatches between parasites and hosts (Rohr et al., 2011). Parasites are also threatened by deliberate attempts to control or eradicate them. In certain circumstances, the extirpation of parasites of public health or veterinary importance can be an unquestionable gain, but control efforts often affect species beyond those initially targeted (Kristensen and Brown, 1999). In other instances, routine veterinary practices can have the unintended effect of eliminating intermediate hosts and thereby interrupt enzootic transmission cycles in species other than those receiving the treatment (Spratt, 1997; Wardhaugh et al., 2001).

**Fig. 1:**
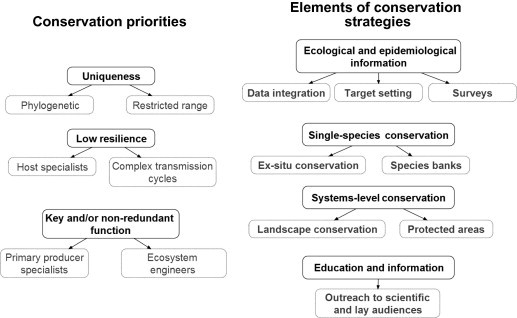
A conceptual framework for parasite conservation. Practitioners should establish conservation priorities (here we show the prioritization categories in Gómez et al., 2012) and design conservation strategies using a combination of interrelated approaches.

Parasites and other associated taxa are threatened not only by direct environmental alteration but are also indirectly affected by all the threats acting upon their hosts (Colwell et al., 2012). Parasites’ dependence on their host populations implies that they face the risk of co-endangerment and co-extinction when hosts decline. As many parasites require a threshold host population size for sustained transmission, some species will be endangered well before this decline is irreversible (Altizer et al., 2007; Powell, 2011). Although such co-extinctions in dependent taxa likely represent the majority of extinction events in this age of unprecedented biodiversity loss (Koh et al., 2004; Dunn et al., 2009), discrepancies remain between the number of documented and expected co-extinctions (Dunn et al., 2009). However, the threat of co-extinction must be carefully evaluated in any parasite conservation assessment. Estimating the extent of the co-extinction threat for specific parasites will depend on detailed understanding of host and parasite ecology, natural history, phylogeny, and key attributes such as host specificity and multi-host life cycles (Gómez et al., 2012; Fig. 1).

Additionally, common conservation strategies for hosts such as captive management, reintroduction, and translocation include broad-spectrum veterinary treatments to limit or prevent parasite transmission (Phillips and Scheck, 1991; Moir et al., 2012). By maintaining disease-free host populations, the likelihood of conservation intervention success (at least in the short term) may be increased at the expense of parasite decline or extinction, especially for parasites of endangered, rare, or spatially restricted hosts (Gómez et al., 2012; Fig. 1). For example, the extinction of the louse *Colpocephalum californici*, is suspected to be associated with the ex-situ veterinary treatment of California condors (Koh et al., 2004). However, such interventions can lead to unanticipated and negative impacts for hosts, including increased susceptibility of hosts to infection following reintroduction or translocation (Gompper and Williams, 1998; Almberg et al., 2012). This suggests that the maintenance of host-parasite relationships in managed wildlife populations can be ultimately beneficial, and points to a critical role for wildlife parasitologists in conservation efforts.

Using data on parasitic helminths of endangered vertebrates, Dobson et al. (2008; see also Gómez et al., 2012) estimated that over 200 species are currently endangered or have become extinct. Mihalca et al. (2011) estimate that 63 hard tick species are currently co-endangered, and that at least one species has recently become extinct. Their work extends previous estimates by Durden and Keirans (1996), in which an additional five tick species were found to be endangered. Colwell et al. (2009) conclude that many oestrid fly species are co-endangered or have been lost through co-extinction or as unintended casualties of widespread antiparasitic drug use. As current estimates of global parasite diversity do not yet incorporate most host taxa, often inadequately sample those taxa that are incorporated, largely exclude microscopic parasitic diversity, and many of the parasite species upon which these estimates are based are more likely to represent clusters of cryptic species (Poulin and Morand, 2004; Angly et al., 2006; Cotterill et al., 2008; Dobson et al., 2008), such estimates are likely to severely underrepresent the true level of threat faced by parasitic diversity (Dunn et al., 2009).

## 4 Should we care?

The loss of parasites might be understood as an unrivaled benefit for free-living biodiversity. Consequently, efforts to conserve parasites must clearly articulate their motivating values. Ethical and aesthetic arguments for conservation apply equally well to parasitic as to free living biodiversity (Gompper and Williams, 1998; Windsor, 1998). Notions of intrinsic value are applicable regardless of trophic strategy, and there is no reason why beauty cannot be found in parasite morphology, behavior, or natural history.

Utilitarian arguments for conservation refer to benefits for human health and wellbeing derived from biodiversity. Perhaps paradoxically, utilitarian values associated with the provision of goods and/or services also apply to parasites. Although parasitism creates direct fitness costs, in some situations infection can indirectly result in fitness benefits that exceed those direct costs (Thomas et al., 2000). For example, in the case of cross-immunity, infection with an enzootic parasite of lower pathogenicity can protect hosts from related emerging pathogens, and some infections can protect hosts against unrelated parasites (Hedges et al., 2008). Some intestinal helminths can bioaccumulate heavy metals, potentially removing significant amounts from the host’s tissues (Sures, 2003, 2004; Pascual and Abollo, 2005). The relevance of parasites in ecosystem organization, and in maintaining baseline ecological dynamics can themselves be considered a service. Other benefits provided by parasites including services related to human health are reviewed by Gómez et al. (2012).

Parasites may additionally aid conservation efforts by providing information about the status of ecosystems. Due to their conspicuous roles in trophic networks, parasites can be indicators of food web structure (Marcogliese, 2004; Lafferty et al., 2006). Studies of parasites can also shed light on the host’s evolutionary and demographic history (Nieberding and Olivieri, 2007), migratory patterns (Killingley, 1980), and help identify host origins (Harris et al., 2013). Finally, parasites can also be effective indicators of ecosystem integrity and human influence (Sasal et al., 2007; Palm and Rückert, 2009), and provide information about host ecology at lower sampling effort and/or cost than those required to survey the hosts directly (Hechinger and Lafferty, 2005; Hechinger et al., 2007; Byers et al., 2011).

## 5 Can we conserve parasites?

Existing measures to conserve or manage parasites are remarkably scarce, despite calls to increase parasite representation in endangered species listing (Dunn et al., 2009; Dunne and Williams, 2009), or other conservation approaches (Windsor, 1995; Perez et al., 2006; Pizzi, 2009). Progress towards the integration of parasites into proposed or existing conservation efforts is likely to require a combination of at least four interrelated elements: (1) improved ecological and epidemiological information, (2) single-species conservation efforts, (3) systems-level conservation efforts, and (4) a widespread perception of parasites as species worthy of conservation efforts (Fig. 1).

*Improved ecological and epidemiological information* will be critical in the conservation or management of host-parasite interactions. Parasite conservation requires setting and maintaining target levels of transmission. Setting such complex targets requires a combination of ecological and epidemiological data that are generally absent for most host-parasite relationships. This knowledge gap has real consequences for our capacity to understand the role of parasites in ecosystems, and hinders our ability to incorporate parasites into wildlife, fisheries, and land management plans. In most cases we lack the knowledge needed to understand if observed epidemiological patterns associated with host population changes are abnormal or merely signs of a restored ecological relationship. Even in the presence of indicators of host conservation success, concurrent increases of infection incidence or parasite loads can be understood as a negative consequence requiring intervention (Wootton et al., 2012). Although not unique for parasitic biodiversity, lack of information with which to set conservation goals is a critical challenge for parasite conservation. Overcoming this gap will require a combination of extensive survey efforts, and integrated biological collections and archival data resources to enable assessments of change in both parasites and host species.

*Single-species conservation measures* strategies established specifically for parasites are extremely scarce. We know of no wildlife parasite with a recovery or management plan, even for those species with extreme host specialization or host geographic restriction. Nevertheless, conservation strategies for specific parasite species are available. Parasites of endangered hosts can be maintained in alternative species in captivity or ex-situ (Vesk et al., 2010). Specimen banks can provide a safeguard against extinction and a source for future reintroductions. However, the requirements for success (e.g. lack of host specialization, culturability), and cost of these strategies restrict their applicability across parasitic taxa (Moir et al., 2012).

*Systems-level conservation interventions* refer to decision-making processes at larger spatial scales, intended to encompass entire ecosystems or landscapes, and include a variety of interventions, from large-scale conservation planning to natural resource management. Conservation as a byproduct of this type of intervention is the predominant *de facto* strategy for parasitic biodiversity, regardless of the fact that the alignment of conservation goals for free-living biodiversity with the conservation of parasites has received scant attention. However, recent studies suggest that systems-level conservation strategies do affect parasite diversity and infection patterns. For example, some protected areas hold a greater diversity and/or abundance of parasitic taxa than unprotected sites (Loot et al., 2005; Lafferty et al., 2008b; Wood et al., 2013). Nevertheless, as protected areas have thus far never been explicitly intended to conserve host-parasite relationships, their placement, design, and management are blind to parasite ecology. Beyond risking the loss of parasitic species, lack of attention to parasite ecology can lead to unintended health costs for protected wildlife. (Ezenwa, 2004; Lebarbenchon et al., 2006).

*Improved perceptions* of parasites may play a key role in driving support for the strategies listed above. In the scientific community, the development of parasite conservation strategies might be hindered by the lack of inclusion of parasites in academic conservation science, their erroneously perceived irrelevance in ecosystem functioning and evolutionary dynamics, and their conspicuous absence in educational materials for conservation biologists (Nichols and Gómez, 2011). From a non-scientific perspective, popular science writing approaches that highlight the positive roles of parasites in ecosystems and the dangers of parasite biodiversity loss may help capture the attention of funding bodies and decision makers (Zimmer, 2000; LaFee, 2006; Zuk, 2007).

Concerted efforts will be required to provide a counterweight to the fact that the conservation of parasites implies the maintenance of morbidity and mortality in wildlife and domestic animals, and the preservation of the zoonotic pool from which many human pathogens come. For those interested in parasite conservation, the possibility of maintaining the causes of diminished wellbeing is a substantial charge. Disease-related human-wildlife conflict is a matter of global concern as in an increasingly interconnected world, these are risks no longer limited to populations living in the edges of the agricultural frontier. Yet, the preponderance of wildlife parasites as emerging pathogens of humans should not prevent parasite conservation research or action. In fact, emerging pathogens are not a random sample of all wildlife parasites (Taylor et al., 2001), and non zoonotic parasites form the bulk of the human disease burden (Kuris, 2012). The necessity to preserve public health need not translate into blanket condemnation of parasites.

Public relations efforts may also be needed within the conservation community. For managers of wildlife and wild areas, the added task of monitoring and maintaining host-parasite relationships can be a diversion of the resources needed to achieve the conservation of the hosts, and our current knowledge is still insufficient to translate generalized warnings against the potentially negative consequences of parasite loss into actionable conservation targets. However, it may be that parasites do not necessarily create an additional management task but rather that maintaining parasite transmission can be an inclusive metric with which to monitor host and ecosystem conservation efforts.

## 6 Conclusion

Recent research has shown that basic assumptions about parasites, their ubiquity, and their relevance need to be reexamined or abandoned. We now know that parasitism may be the most widespread animal trophic strategy. Of the 42 broadly recognized phyla, 31 are entirely or predominantly parasitic, and most others have multiple parasitic clades (Poulin and Morand, 2000; de Meeûs and Renaud, 2002). Parasitic helminths alone are about twice as speciose as their vertebrate hosts (Poulin and Morand, 2000; Dobson et al., 2008). We also know that parasitic biomass can be substantial, and often equal or greater than that of other groups. Finally, we have learned that parasites play critical roles in ecological and evolutionary processes, and that infection may drive ecosystem services.

Nevertheless, parasite conservation can be a challenging proposition. Available evidence strongly indicates that many parasite species are endangered and that their loss can substantially affect the normal functioning of ecosystems, represent disproportionate losses of evolutionary potential, and potentially affect the long-term persistence of their hosts (Gompper and Williams, 1998; Dunn et al., 2009). Yet we remain generally unable to quantify the costs associated with parasite loss. Consequently, our ability to contextualize the need for parasite conservation among competing demands is limited. Will dedicated efforts to include parasites in conservation strategies result in increased success in achieving overarching conservation goals? Will conserving parasites facilitate the long-term persistence of hosts? Answering these questions will help us decide how much of the scarce attention and resources available for conservation should be invested in parasites.

A more inclusive consideration of parasitic biodiversity suggests that all of the arguments espoused to conserve free-living species apply equally well to parasites. This broader view suggests that the ecological relationships between hosts and parasites are relevant and therefore that the roles of wildlife parasitologists may warrant frequent involvement in the science and practice of parasite conservation. Much needs to be done before wildlife parasites become intentional targets of conservation action, but ignoring parasites in efforts to conserve biodiversity means neglecting critical components of both the patterns and the processes that form natural ecosystems.
